# Nonnative Energetic Frustrations in Protein Folding at Residual Level: A Simulation Study of Homologous Immunoglobulin-like *β*-Sandwich Proteins

**DOI:** 10.3390/ijms19051515

**Published:** 2018-05-18

**Authors:** Yunxiang Sun, Feng Ding, Dengming Ming

**Affiliations:** 1 College of Biotechnology and Pharmaceutical Engineering, Nanjing Tech University, 30 Puzhu South Road, Nanjing 211816, China; yunxias@clemson.edu; 2 Department of Physics and Astronomy, Clemson University, Clemson, SC 29634, USA; fding@clemson.edu

**Keywords:** nonnative energetic frustration, non-native contact, frustrated Gō-like model, *β*-sandwich protein, hydrophilic-hydrophobic mutation

## Abstract

Nonnative interactions cause energetic frustrations in protein folding and were found to dominate key events in folding intermediates. However, systematically characterizing energetic frustrations that are caused by nonnative intra-residue interactions at residual resolution is still lacking. Recently, we studied the folding of a set of homologous all-*α* proteins and found that nonnative-contact-based energetic frustrations are highly correlated to topology of the protein native-contact network. Here, we studied the folding of nine homologous immunoglobulin-like (Ig-like) *β*-sandwich proteins, and examined nonnative-contact-based energetic frustrations Gō-like model. Our calculations showed that nonnative-interaction-based energetic frustrations in *β*-sandwich proteins are much more complicated than those in all-α proteins, and they exhibit highly heterogeneous effects on the folding of secondary structures. Further, the nonnative interactions introduced distinct correlations in the folding of different folding-patches of *β*-sandwich proteins. Taken together, a strong interplay might exist between nonnative-interaction energetic frustrations and the protein native-contact networks, which ensures that *β*-sandwich domains adopt a common folding mechanism.

## 1. Introduction

Most nascent polypeptide chains undergo a series of folding events before they acquire the compact biologically functioning three-dimensional (3D) conformations. The dynamics of intra-residue contacts defines the details of protein folding kinetics, which finally leads a protein to its native structure. Accurate modeling residue interactions in protein folding processes is still a challenging task in the community, although much progress had been made both experimentally and theoretically [1]. Specifically, during protein folding, some residues come close to each other, and then stay together and form the so-called native contacts in the native structures, while others only form transient contacts—called non-native contacts, which are separated in the final structures. Both experimental data and theoretical simulations have suggested that native-contacts play essential roles in determining both the protein folding kinetics and the native structures [2,3,4,5]. At the same time, minimally frustrated models of protein folding were developed, where non-native intra-residue interactions are systematically suppressed [6,7,8,9]. These models lead to the success of the free energy landscape theory in solving the kinetic paradox of protein folding problems [10,11,12,13,14], and in interpreting a variety of experimental studies, such as the single molecule pulling [15,16,17]. According to the nucleation mechanism of protein folding, the formation of a few key native contacts in the polypeptide chain can start a series of down-hill like conformation changes towards the protein native states, without being trapped in any intermediate states at a local minimum.

Although native-contacts dominate the folding kinetics of many proteins, recent experiments and theoretical calculations found that non-native contacts also play a non-trivial role in protein folding [18,19,20,21,22,23,24,25]. Many non-native contacts are found in intermediate states of protein folding between the unfolded state and the fully folded native state. For example, non-native hydrogen bonding was identified between strand *β*_1_ and strand *β*_2_, stabilizing an intermediate state of a SH3 domain under the native condition, using a relaxation dispersion nuclear magnetic resonance (RDNMR) spectroscopy technique [26]. The formation of nonnative contacts may either speed up the folding by stabilizing transition states and lowering the folding free energy barrier [18,27,28], or slow it down by trapping the molecules in local minimums in the free energy landscape [29]. Thus, nonnative contacts can have different effects on protein folding, depending on where they take place. Recent years saw many computational simulations of nonnative interactions in protein folding [30,31,32,33,34,35,36,37,38,39], of which the nonnative hydrophobic interactions (formed between hydrophobic residues) were studied very extensively. Most of these studies were performed based on some frustration model, in which non-native residual interactions were added as a perturbation to protein folding.

Two types of frustrations for protein folding had been extensively studied, namely, the topological frustration and the energetic frustration [40]. Topological frustration results from the fact that the protein usually holds a particular 3D shape of native fold, and residual excluded volumes prevent the protein from sampling certain area of configuration space. Such steric hindrances thus cause some parts of the protein less likely to contact one another, and lead to the folding frustration. For example, in terms of protein shape, proteins with irregular topologies often have much more difficulties to fold than do the little symmetric ones [41], and thus experience more frustrations in folding. Energetic frustration is directly associated with the incorrect intra-residue interactions or non-native contacts that are not presented in the protein native structures. Although natural selection helps to minimize energetic frustration by optimizing amino acid sequence of the protein (especially for smaller and globular proteins), certain number of energetic frustrations, here called the non-native-contact frustration, may still remain. Non-native-contact frustrations have been studied by analyzing the so-called protein transition state ensembles (TSEs). TSEs delineate the profile maxima on reaction paths that connect the fully folded native-like states and the unfolded states in protein folding [42]. Sutto et al. used TSEs to identify local frustrations, mostly energetic frustrations, in the on-pathway intermediates of the four-helix Im7 protein using an all-atom AMW model [31]. Shea et al. analyzed protein folding transition states to study the degree of energetic frustration in the folding of a 46-mer four standard *β*-barrel model protein [43]. Recently, Chung et al. examined energetic frustrations in a designed *α*-helical protein, and identified specific intra-residue contacts that decreased the folding rate [24]. In summary, protein folding frustrations can involve both structural and dynamic factors due to changes in topologies and amino acid sequences of studied proteins.

Recently, we studied the energetic frustrations of five homologous four-helical Im9 proteins at the residual level [44]. The selected proteins share the same 3D topological structures and thus have the same native contact networks, but they have a single hydrophilic-to-hydrophobic mutual mutation. Our studies characterized energetic frustrations in the folding of all-*α* proteins, and clearly showed that energetic frustrations are very sensitive to the local structural environments where non-natice contacts are introduced. Specifically, we found that frustrations at regions of dense native contacts cause large perturbations to the folding of all-*α* proteins. The importance of local native contact density was also emphasized in a recent protein folding study of bacterial Immunity proteins Im7/Im9 whose secondary structure component are also all *α*-helices [36]. When considering the fundamental difference of the 3D topologies between all-*β* sandwich proteins and all-*α* proteins, it is interesting (1) to study the effects of energetic frustrations on the folding of all-*β* proteins, and (2) to investigate the interplay between the energetic frustration and the local native contact density in these proteins.

In this paper, we examined energetic frustrations at residue level, via *φ*-value analysis of TSEs, of five homologous *β*-sandwich immunoglobulin-like proteins and four artificially mutated structures, using a similar strategy as that in reference [44]. The all-*β* sandwich proteins were selected from the same domain entry in the structural classification of proteins (SCOP) [45], and shared the same 3D topologies and almost the same amino acid sequences except one or two site-mutations. The structural similarity among selected structures minimized the difference in their topological frustrations, thus enabling us to focus on the effects of energetic frustrations. Previous studies showed that, unlike all-*α* proteins, all-*β* proteins usually share similar folding pathways [46]. Hence, we might expect all-*β* proteins have a different energetic frustration mechanism when compared with that in all-*α* proteins. Our calculations showed that energetic frustrations in the examined *β*-proteins are highly heterogeneous, depending on local environments where nonnative intra-residue contacts are introduced. Specifically, we found a larger distribution of energetic frustrations in the folding of the all-beta proteins, as compared to that in all-*α* proteins. We ascribed this difference to the particular topology of all-*β* proteins, where no native contact center exists and the local native contact density is more evenly distributed.

## 2. Results and Discussions

**Figure 1:**
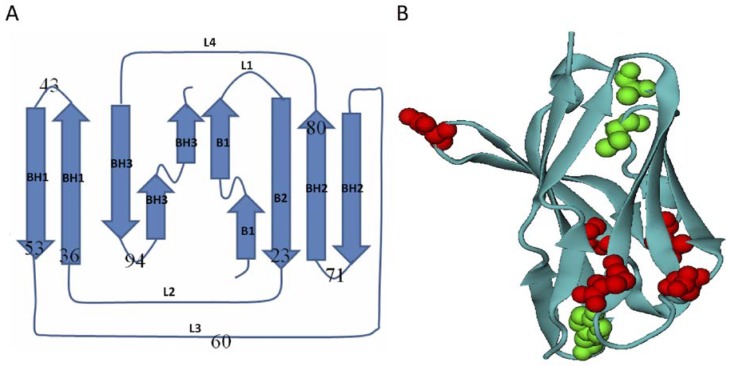
The topology of selected Ig-like *β*-sandwich domains and the location of mutations. (**A**) Topology of Ig-like *β*-sandwich domains. The secondary structures are *β*-strand **B1**: residue 1-12, linker **L1**: residue 13-15, **B2**: 16-24, **L2**: 25-35, *β*-hairpin **BH1**: 36-43 & 44-51, **L3**: 52-63, **BH2**: 64-70 & 71-80, **L4**: 81-85, and *β*-hairpin **BH3**: 86-96 & 97-110. The *β*-sheet region was identified using the DSSP program [49]. (**B**) The tertiary structure of Ig-like *β*-sandwich domains with the mutations highlighted with solid spheres: read ones are hydrophilic-to-hydrophobic mutations while green ones are the reverse mutations. The is figure was prepared using VMD software [50].

The selected structures are mutually superposed with a small *C_α_* root-mean-square-RMSD that is between 0.4 and 0.8 Å (see Table 1), thus minimizing topological frustration differences among them and highlighting energetic frustrations caused by different amino-acid replacements. Ig-like *β*-sandwich domains comprise of eight *β*-stands (B1-B8), of which the latter six ones formed three *β*-hairpins (BH1-BH3) (see Figure 1). The second *β*-stand B2 and the second *β*-hairpins BH2 comprise the first *β*-pleated sheet, and B1, BH1, and BH3 form the second *β*-sheet. These two *β*-sheets are tightly packed through linkers L2 at the bottom and L4 on the top. For clarity, we named each domain after its dominant hydrophilic-hydrophobic mutation (see the caption in Figure 1). Mutations are divided into two groups: (1) the hydrophilic-to-hydrophobic mutation type: R23A, N36I, D43A, T53A, D71A, and (2) the hydrophobic-to-hydrophilic mutation type: V60T, A80G, Y94D. Besides these mutations, homotypic mutations also exist in three structures: domain R23A has an additional mutation of T21K, domain N36I has T47S, A57V, I77V, and another hydrophilic-hydrophobic mutation Q1F, and domain Y94D has N55K, W98L. These systems are simulated using both the conventional coarse-grained Gō-like model and the frustrated model. To examine the perturbation of these mutations on protein folding, we calculated residual *ϕ*-values and intra-residue contact maps, and thus determined the effect of energetic frustration as a change in residual *ϕ*-value and intra-residue contact [47,48].

### 2.1. Transition State Ensembles of Ig-like β-Sandwich Folds are Sensitive To the Energetic Frustration Mutation Centers

**Figure 2:**
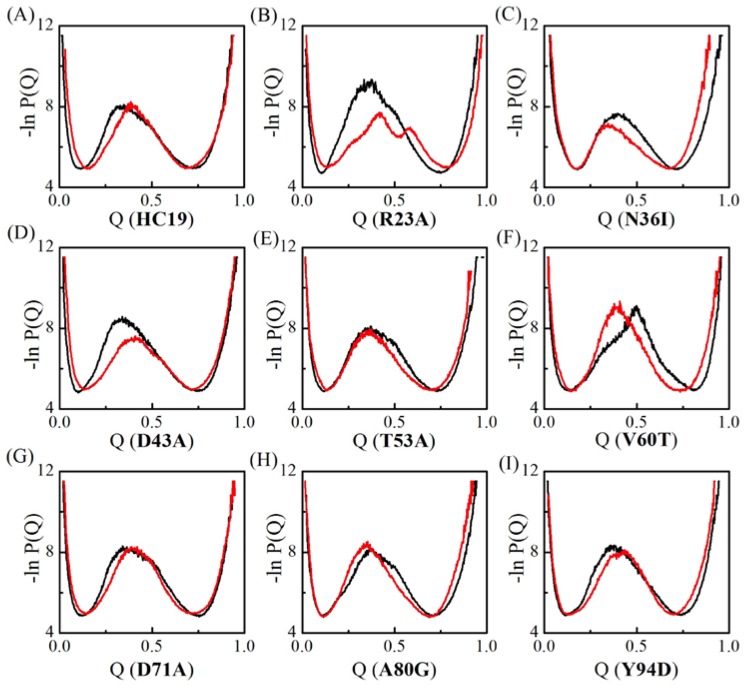
Apparent folding free energy changes for the nine Ig-like *β*-sandwich domains. Black stands for the conventional Gō-like minimal model and red for the frustration model. Different mutants are indicated by IDs (see caption of Table 1) inside the parenthesis of x-coordinate titles for A-I.

The conformations in TSEs of Ig-like *β*-sandwich folds are sorted with respect to Q value—the total number of native contacts that are preserved in given conformation normalized by the total number of native contacts found in native states. Q value varies between 0 and 1, corresponding to the fully unfolded states and the fully folded native states, respectively. The apparent free energy of the system in terms of Q-value can be derived as the negative log of the conformation distribution probability (−lnP(Q)). In VTF simulations, an averaged collapse temperature ${\bar{T}}_{\theta }$ is used in determining the free energy temperature factor $k_{B}{\bar{T}}_{\theta }$. Figure 2 compares the apparent “free energy” landscapes for the two models. The changes in free energy landscape are recognized as the shift of the location of central energy barrier, which corresponds to the TSEs. Essentially, two groups exist: one group takes right-shift with the free energy profile shifting to the high Q-value end, indicating more native contacts that are preserved in TSEs. The second one takes a left-shit with less native contacts in TSEs. Five domains **HC19**, **R23A**, **D43A**, **D71A**, and **Y94D** have a right-shifted free-energy change, a common change been observed in the folding of Im9 all-*α* proteins [51]. However, different from that in all-*α* proteins, left-shifted free-energy change also happened in **N36I**, **T53A**, **V60T**, and **A80G**, indicating nonnative-contact energetic frustrations destroys some native contacts in TSEs. Further, significant lower barriers were observed in domains **R23A** and **D43A**, which is different from that in all-*α* proteins where energetic frustrations tend not to decrease the barriers [51]. Wolyens and colleagues examined the folding of three typical proteins, namely the all-*α*, *α*/*β* and all-*β* proteins [46], and showed that the introduction of the native energetic frustration essentially tends to lower the free-energy barrier. When compared with native energetic frustrations, the nonnative ones in all-*β* structures can either increase or decrease the barrier in significant level, and they can either facilitate or impair the formation of native-contacts in TSEs depending the introduced nonnative-contact locations.

### 2.2. Energetic Frustrations Have Highly Heterogeneous Effects on the Folding of Ig-like *β*-Sandwich folds

When considering that all examined structures share the same tertiary structure (see Table 1), we might safely ascribe the difference in residual ϕ-value changes to the addition or elimination of nonnative-hydrophobic-contact energetic frustrations that are caused by the mutations. Since most of the mutations only involve a single hydrophilic-hydrophobic mutation among the examined structures (except domain **N36I**), and we can further correlate the effects of energetic frustrations with the local environment where mutations happen.

**Figure 3:**
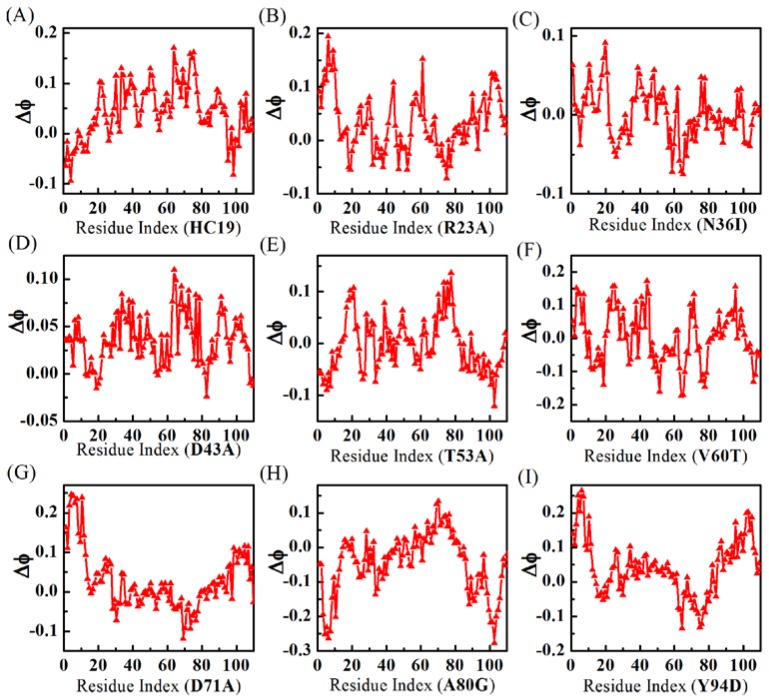
*ϕ*-value changes show that energetic frustrations are heavily dependent on mutation locations in the *β*-sandwich structures. Different mutants are indicated by IDs (see caption of Table 1) inside the parenthesis of x-coordinate titles for A-I.

Figure 3 shows residual *ϕ*-value-changes derived from the two models. The absolute *ϕ*-values (see Supplementary Figure S2) fall short of experimental measurements [52] (see a comparison with experimental *ϕ*-values in Supplementary Figure S3), possibly because the simulations were done in vacuum while experiment measurements were performed in water. In this sense, the relative *ϕ*-values make more sense than the absolute values. When compared with all-*α* Im9 domains, the calculations show that all-*β* domains have more irregular residual *ϕ*-value changes upon energetic frustrations. For example, no significant residual *ϕ*-value change is found in both domains **N36I** and **T53A**, indicating the effects of energetic frustrations in these proteins are ignorable. For **V60T**, the residual *ϕ*-values decrease in BH2 is balanced by increase in B1, leaving an averaged *ϕ*-value almost unchanged. In **HC19**, significant *ϕ*-value increases are found in linker L2, *β*-hairpins BH2, and BH3, while a small decrease happens in the first *β*-strand B1. **R23A** has detectable *ϕ*-value increase in B1, BH3, and C-strand of L3. **D43A** has small increases in B1, L2, N-strand of BH1, and N-strand of BH2. **D71A** has larger *ϕ*-value increases in B1 and detectable decreases in BH2. In **A80G**, residual *ϕ*-values become smaller in B1 and BH3. **Y94D** obtains significant *ϕ*-value increases in B1 and BH3, and small decrease in BH2.

Taken together, both introducing (in hydrophilic-to-hydrophobic mutant) and eliminating (in hydrophobic-to-hydrophilic mutant) of energetic frustrations can result in complicated fluctuations to the folding of most secondary structures in *β*-sandwich structures.

**Figure 4:**
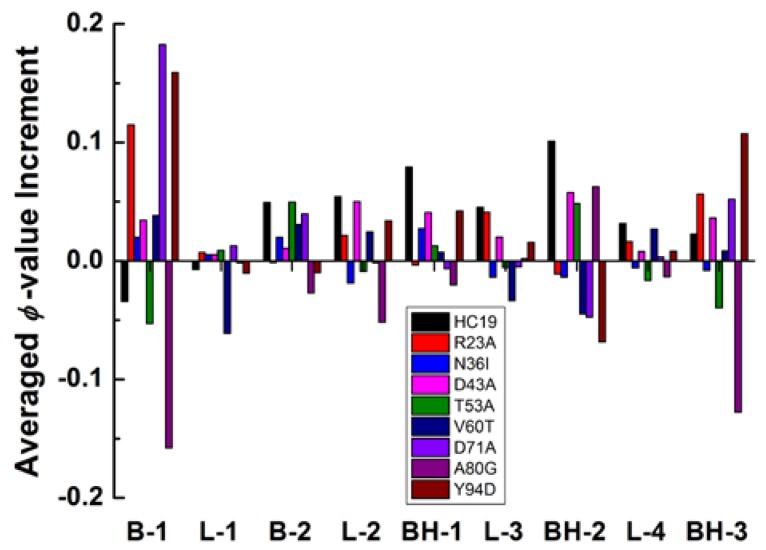
Averaged *ϕ*-value changes as a function of secondary structures for the nine *β*-sandwich domains.

The heterogeneity of energetic frustrations can be appreciated more clearly with changes of averaged *ϕ*-values of secondary structures (Figure 4). The largest changes happen in B1 and BH3, the two elements occupy the N- and C-termini of the structure, respectively. These two secondary structures have a significant *ϕ*-value increase for five of the nine studied proteins and a detectable *ϕ*-value decrease in **A80G** and **T53A**. The only inconsistence occurs in HC19 where B1 has lower *ϕ*-values while BH3 has larger ones. Topologically, good packing between B1 and BH3 should be critically important for *β*-sandwich structure to reach its native state. The second *β*-hairpin BH2 also has significant change in residual *ϕ*-value distribution: significant increase in **HC19**, **D43A**, **T53A**, and **A80G** and moderate-to-weak decrease in other cases. More interestingly, BH2 seems to do the reverse as B1 and BH3 in altering their *ϕ*-values. For example, in **R23A**, **N36I**, **D71A**, **Y94D**, B1, and BH3 gain larger *ϕ*-values, while BH2 has smaller ones. In **T53A** and **A80G**, smaller *ϕ*-values are found in B1 and BH3 while larger ones found in BH2. The second *β*-strand and the first *β*-hairpin show less *ϕ*-value perturbations as compared with **B1** and **BH2/3**. All of the linkers except **L2** have ignorable *ϕ*-value changes upon energetic frustration. Even for **L2**—the linker connects the two *β*-sheets at the bottom—only a moderate-to-weak change is observed. To summarize, the heterogeneity of energetic frustration in the folding of *β*-sandwich proteins lies in two folds: a single site-mutation might cause fundamentally different perturbation to the overall folding status (see the difference between **HC19** and **A80G**), indicating that energetic frustration is highly site-sensitive to the local environment; secondary structure elements may have different responses to the same energetic frustration, as revealed by *ϕ*-value changes in BH2 and those in BH3 and B1.

It is interesting to compare the energetic frustration effects in all *β*-sandwich domains with those in all-*α* Im9 proteins. Energetic frustration increases residual *ϕ*-values for all of the examined all-*α* domains (see Table 2 in reference [51]), of which the largest increment happens in D51A whose mutation occurs in the center of the native-contact network. In this center area, a hydrophilic-to-hydrophobic mutation facilitates the formation of native-contacts among residues from the three surrounding long helices. However, in all *β*-sandwich structures, native contacts are much evenly distributed within the structures, and lack a center area where a relatively large energetic frustration may happen just like that in the all-*α* **D51A** domain [51]. Thus, energetic frustrations of *β*-sandwich structures show little dependence on the mutation locations. In a recent study on a weakly frustrated *β*-structure of *Tm*CSP [34], Onuchic and colleagues found that many residues have lower *ϕ*-value increase, and high values decrease, upon the addition of frustration. Essentially, this is consistent with the heterogeneity of *ϕ*-value change, as revealed in Figure 3, where most residues exhibit small *ϕ*-value increase, and a few residues have high-value decrease (such as in **A80G**).

### 2.3. Energetic Frustration Alter the Folding Consistency Between the Folding Patches in *β*-Sandwich Structures

The enclosed topology structure of *β*-sandwich domains requires a concomitant formation of the secondary structure and then the tertiary structure in folding, resulted in a typical nucleation-condensation folding mechanism [53,54]. The folding contact-map of *β*-sandwich structures calculated from TSEs shows similar secondary-structure interactions during protein folding (see detailed comparison in supplementary Figure S4). The contact map in the upper left triangle represents energetic frustrated simulation, which is compared with that in the bottom right triangle that is derived from the conventional minimal model. The matrix diagonal records intensive interactions between intermediately neighboring anti-parallel *β*-strands, as following: (B1, B2), (BH1, BH1), (BH2, BH2), (BH3, BH3). Moderate secondary-structure-contacts appear in the bottom right and upper left area, including anti-parallel *β*-strands (N-strand of BH1, N-strand of BH3), (B2, N-strand of BH2) and parallel *β*-strands (B1, C-strand of BH3). The strongest contacts happen in the N- and C-strands of BH3, especially at its central-turn. The second strongest interactions occur between BH1 and BH3 in the middle of the second *β*-sheet. The nine proteins share similar contact maps highlighted by above-mentioned *β*-strands contact-patches. Thus, they are likely to have similar overall TSEs during protein folding and share the same folding mechanism, which is consistent with previous studies in that immunoglobulin-like *β*-sandwich proteins tend to share a common nucleation-condensation folding pathway.

**Figure 5:**
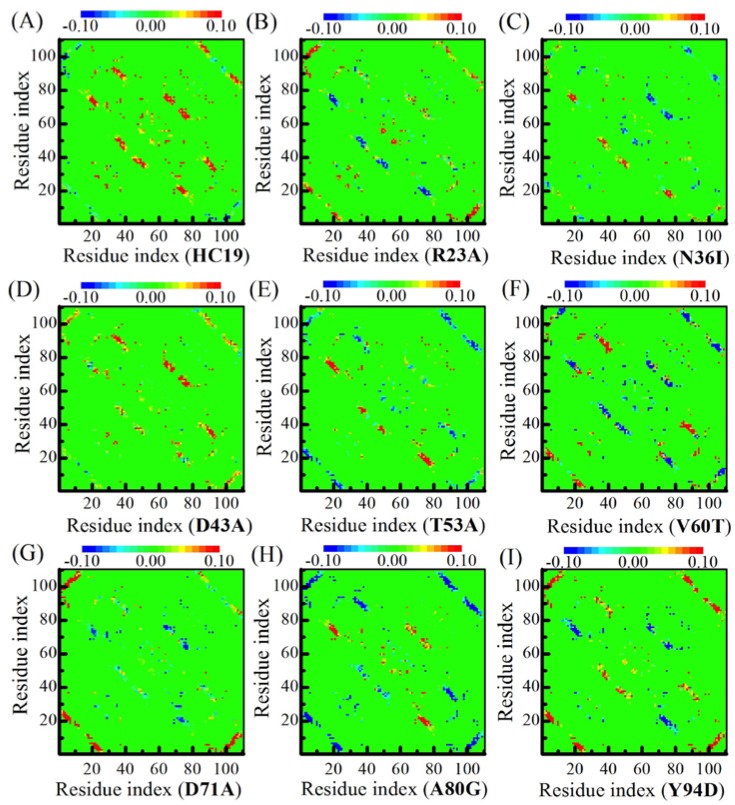
The contact difference maps for the nine *β*-sandwich domains. The difference of contact map is associated with the local environments of site-mutations. Different mutants are indicated by IDs (see caption of Table 1) inside the parenthesis of x-coordinate titles for A-I.

To examine the effects of energetic frustrations on the folding pathway of *β*-sandwich proteins, we calculated the contact-map differences by subtracting the upper triangle element from its lower triangle symmetric counterpart (see Figure 5). The color of matrix elements represents the contact-tendency between two residues: red for enhanced contact and blue for weakened contact. The heterogeneous effects of energetic frustrations are read from the inconsistent distributions of red and blue patches in the folding of nine domains. The most prominent changes occur in *β*-strand pairs along the diagonal, including the three *β*-hairpins and the initial pair of (B1, B2). We noticed that in a single patch the color distribution is pretty even: they are either red or blue, indicating that the collective folding/unfolding exists in the patches formed by neighboring *β*-strand pairs. In this sense, we call these *β*-strand pairs the folding patches.

Our calculations show that folding patches have different correlation with one another in response to energetic frustrations, depending on patch elements and the locations where mutations happen. For example, BH1 and BH2 (the two patches in the center of the diagonal) have the same folding propensity in **HC19** and **V60T**, but take the reverse tendencies in **N36I** and **Y94D**. Another two patches, B1-B2 and BH3, at the two ends of the diagonal, show a different response: both increase folding tendency in mutations **R23A**, **T53A**, **A80G**, and **Y94D**, but decrease folding tendency in **V60T**. The two elements in the patch pair, B1-B2 and B1-C-strand of BH3, show similar folding tendency correlation, as does the patch pair B1-B2 and BH3. It is interesting to examine the site-mutation between **HC19** and **V60T**: energetic frustrations enhance the folding in all of the three *β*-hairpins in **HC19**, but weaken the folding in **V60T**. These results suggest that energetic frustrations bring heterogeneous effects on the local folding status in *β*-sandwich structures. At the same time, energetic frustrations generate alternative correlations between folding-patches, which are sensitive on local environment of mutations.

## 3. Methods

### 3.1. Homologous Domains of Immunoglobulin-Like β-Sandwich Structures

**Figure 6:**
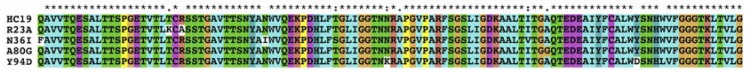
Sequence alignment of the five selected Ig-like *β*-sandwich domains selected from SCOP. The abbreviations read **HC19:** d1gigl1, **R23A**: d4a6yl1, **N36I**: d1etzl1, **A80G**: d2y06l1, **Y94D**: d3ks0l1. Additional four structures are derived from **HC19** by artificial single-site mutation at site D43, T53, V60 and D71, and named as **D43A**, **T53A**, **V60T**, **D71A**, respectively.

The key of this study hinges on comparing the folding of homologous all-*β* proteins that share very similar 3D topology structures and amino acid sequences. Although the Ig-like *β*-sandwich fold had been subjected to wide studies as a model of all-*β* system in protein folding [52,53,55,56], the examined proteins in published works vary a lot in either structure or sequences. We selected protein candidates from the immunoglobulin-like domain entry of b.1.1.1 in the SCOP database [45,57]. Five Ig-like *β*-sandwich domains d1gigl1, d3ks0l1, d4a6yl1, d2y06l1, d1etzl1 (PDB codes 1GIG, 3KS0, 4A6Y, 2Y06, 1ETZ, respectively) were selected, and they have almost identical amino acid sequence except a single dominant hydrophilic-to-hydrophobic mutual mutation in a total 110 residues (Figure 6). The mutations were recorded based on the alignment with the protein domain d1gig1l (see caption of Figure 6). These domains share the same complex Greek-key topology, and their 3D structures are described as two tightly packed *β*-sheets packing (Figure 1a). The mutual root-mean-square deviations (RMSDs) between selected structures are between 0.4 and 0.8 Å, which minimized topological frustration due to the structure differences among studied proteins. Unlike the topology of all-*α* homologous Im9 domains where relatively dense native contacts exist in the center [51], here, in the *β*-sandwich structures, native contact distribution is fairly even. It is at the *β*-strand-connection areas where native contacts fluctuate relatively larger, and, in these areas, selected structure exhibit most of the mutual mutations (Figure 1b). Based on this observation, four additional single-site mutants were introduced artificially at different connection-areas so as to explore energetic frustration effects at these new positions. Table 1 summarized the difference between the nine studied all-*β* structures and the mutations. For clarity, each examined domain is renamed after their representative mutation as listed in the caption of Figure 6.

### 3.2. Energetic Frustration Model

The details of protein folding simulations had been described in the previous protein folding studies of Im9 all-*α* domains [51], here we only summarize a few key aspects of the calculations. The minimal frustrated protein folding was performed with an alpha carbon based coarse-grained Gō-like model, whose driving force is determined by the native topology of the studied proteins [58,59,60,61,62,63]. In Gō-like model the potential energy has the following form,

(1) $$U=\overset{N-1}{\underset{i=1}{\sum }}\frac{K_{b}}{2}{\left(r_{i}-r_{i,0}\right)}^{2}+\overset{N-2}{\underset{i=1}{\sum }}\frac{K_{\theta }}{2}{\left(\theta_{i}-\theta_{i,0}\right)}^{2}+\overset{N-3}{\underset{i=1}{\sum }}\frac{K_{\phi }}{2}{\left(1-\cos\left(2\phi_{i}-\frac{\pi }{2}\right)\right)}^{2}\;+\overset{NC}{\underset{i<j-3}{\sum }}\epsilon_{ij}\left[5{\left(\frac{r_{ij,0}}{r_{ij}}\right)}^{12}-6{\left(\frac{r_{ij,0}}{r_{ij}}\right)}^{10}\right]+\overset{NNC}{\underset{i<j-3}{\sum }}\epsilon_{ij}{\left(\frac{C}{r_{ij}}\right)}^{12}$$

where r, θ, ϕ represent instantaneous bond lengths, bond angles and dihedral angles respectively, and the subscript “0” stands for quantities measured in the native state configurations. The upper limit “*NC*” stands for native contacts and it indicates summation over all intra-residue contacts found in native structures, and “NNC” for nonnative contacts and summation over intra-residue pairs that are not directly in contact in the native state. Two residues are determined to form native contact if their minimum atom distance is less than a cutoff value of 5.5 Å in the native conformation. In a transition state, we define that a native contact holds their contact state only when the two C_α_ distance satisfies $r_{ij}/r_{ij,0}\leq 1.2$. Parameters for the model are $K_{b}=200\epsilon_{0}{Å}^{-2}$, $K_{\theta }=40\epsilon_{0}{\mathrm{rad}}^{-2}$, $K_{\phi }=0.3\epsilon_{0}$, and C=4Å. An absolute energy value of $\epsilon_{0}=1.89Kcal\cdot mol^{-1}$ was used following Ref [58] by assuming a folding temperature *T* = 350K for protein G B1 domain. To distinguish different residue pairs, an MJ-flavored coefficient is assigned to a native contact so that the contact energy has the form of $\epsilon_{ij}=\epsilon_{ij}^{MJ}\epsilon_{0}$, where $\epsilon_{ij}^{MJ}$ is proportional to the knowledge-based intra-residue contact energies [64,65,66] and has been normalized by setting the averaged ${\bar{\epsilon }}_{ij}=0.18\epsilon_{0}$. Now, energetic frustrations are introduced by nonnative hydrophobic intra-residue contacts to the minimal frustration model, as following:

(2) $$U_{\mathrm{frustration}}=\overset{NNC}{\underset{i<j-3}{\sum }}\sigma_{ij}\epsilon_{ij}\left[5{\left(\frac{C_{f}}{r_{ij}}\right)}^{12}-6{\left(\frac{C_{f}}{r_{ij}}\right)}^{10}\right]$$

where

$$\sigma_{ij}=\{\begin{array}{cc} 0.5 & \mathrm{if}\;\mathrm{both}\;\mathrm{residue}\;i\;\mathrm{and}\;j\;\mathrm{are}\;\mathrm{hydrophobic},\;\\ 0 & \mathrm{else} \end{array}$$

and $C_{f}=5.5Å$. The protein folding simulations were then performed with the Langevin dynamics scheme, using a time step Δt=0.007τ and a high friction coefficient β=0.2/τ. Here, τ is the characteristic time of the system $\tau_{0}=l_{0}{\left(m/\varepsilon_{0}\right)}^{1/2}$ which is 1.47ps when setting an average residue mass *m* of 119 a.m.u., and an average distance of $l_{0}$ = 3.8Å between adjacent C_α_ atoms. Choosing σ=0.5 is a compromise between the folding efficiency (folding/unfolding transition events dramatically decrease with σ) and the folding frustration (which increases with σ). We noticed that recently a different Guassian type function was also used in the study of energetic frustrations in the Fyn SH3 domain folding [35]. 

### 3.3. The Variable Temperature Protein Folding Simulation

The protein folding simulations is usually carried out at the collapse temperature $T_{\theta }$ at which the calculated specific heat of the system reaches a maximum and the system experiences repeatedly transition between folded and unfolded states [60,61]. However, finding specific heat maxima can be time consuming, especially for large systems. In literature, some calibrated Replica-Exchange protocol was developed for this task [67,68,69,70]. Here, we developed a variable temperature folding (VTF) simulation method, as was used in a previous study [51]. The method is based on the observation that, at collapse temperature, a fast-folding small protein may frequently shift between folded and unfolded states, and thus has equal opportunity to sample either of the two states. In the protein folding free energy landscape, this scenario is featured with a highly-raised barrier in the middle of the two valleys, corresponding to the folded and unfolded states, respectively [30,43]. The VTF method skips the step of determining $T_{\theta }$, instead, it continuously increases or decreases the simulation temperature, thus enforce the system to frequently change its states between folded and unfolded states, ensuring that the system has equal chance to sample either states. Specifically, in VIF calculations, Langevin dynamics simulation is firstly performed for *N_T_* steps with an initial guess collapse temperature *T*. Then, the up-to-now trajectory is collected and its snapshots are grouped based on the number of the total native-contacts (or Q number) of conformation, and a probability density function (PDF) of native-contact number is determined using a histogram method. Usually, when *T* is close to the true collapse temperature *T_θ_*, two peaks will appear in the PDF profile, of which one peak, called the non-native peak, corresponds to smaller Q and denotes the unfolded states, and the other one, which is called native peak, corresponds to larger Q. Next, the simulation temperature *T* is slightly changed by a small value of Δ*T* as following: *T* = *T* − Δ*T* if the nonnative peak is higher than the native peak, or *T* = *T* + Δ*T* when the reverse applies (*T* is randomly updated when the two peaks have the same height). This procedure is kept on going when the trajectory reaches a satisfactory length. When compared with conventional fixed-temperature folding simulations, VTF needs only a single lengthy trajectory for the folding simulation of one protein. In present studies, a typical simulation produces a trajectory that spans 3 µs or has 2 × 10^9^ steps and contains 2 × 10^5^ conformations. Here we set Δ*T* = 0.002 (which is about 0.5%*T_θ_*) and $N_{T}=2\times 10^{7}$, thus a trajectory includes 100 times of temperature updating. VTF simulation enhance the transition between the folding and unfolding states, thus helping to create a large sample of the transition state ensemble (see Figure S1).

### 3.4. φ-Value Analysis and Contact Maps

The first quantity to characterize protein folding states is the total number (Q) of native contacts, preserved in given states. Describing the statistical distribution of the protein configurations with respect to Q-value results in the protein folding free energy landscape, in which the folded and unfolded states appear as two separated peaks. TSEs sample confirmations in between the two peaks. Specifically, transition state conformations were collected whose Q-values fall in the valley area (Q_1_, Q_2_), where Q_1_ is the mean between the Q-value of Valley and that of the unfolded-state peak, and Q_2_ is the mean between Valley and the folded-state peak [42,71,72,73].

The Q-value along, however, cannot fully characterizes the TSEs, since for that same Q-value, the local native-/non-native-contacts may be different from one protein to another. It is the detailed native-/non-native -contacts that determine the protein folding mechanism.

For this sake, *φ*-value analysis had been introduced to measures the perturbations of local residual level native-contacts in both experimental [72] and theoretical studies [74]. *φ*-value compares native-contacts that are formed at a given residue in unfolded and folded states, which is defined as following [4,75,76],

(3) $$\phi_{i}=\frac{\langle N_{i}\rangle {}^{\mathrm{TSE}}}{N_{i}^{\mathrm{nat}}}$$

where *N_i_* is the number of native-contacts preserved in *i*th residue, the denominator is the number in the native state and the numerator the averaged number in TSEs. $\phi_{i}=1$ when residue *i* has exactly the same number of native contacts in TSEs, as it does in the native state, indicating that TSEs preserve full folded state at this residue. $\phi_{i}=0$ indicates that TSEs fall in a fully unfolded state. *φ*-values tell the details of local folding states at residue resolution, thus being able to interpret the consequence of energetic frustrations that are introduced by nonnative hydrophobic intra-residue interactions.

A protein conformation can be interpreted with a two-dimensional *N* × *N* square distance matrix whose element *d_ij_* is the distance between residues *i* and *j*. This matrix is then transferred into the so-called contact-map when the element values replaced by 0 or 1, according to the following equation,

(4a) $$a_{ij}=\{\begin{array}{cc} 1 & \mathrm{if}\;d_{ij}<r_{c}\\ 0 & \mathrm{else} \end{array}$$

where $r_{c}$ is the distance cutoff with a value between 7 to 15 Å. Contact map is a simple two-dimensional representation of a protein’s tertiary structure, thus it is particularly useful to monitor the changes of secondary-structure elements through a large-scaled calculation. In the contact-map, a band along the main diagonal usually stands for an *α*-helix fragment whose contacts are formed among sequentially neighboring residues. On the other hand, *β*-sheet structures may be recognized as bands either parallel to (for parallel *β*-sheet) or perpendicular to the diagonal (for anti-parallel *β*-sheet), and the band thickness tells the number of strands within a *β*-sheet. Contact-map had been broadly used in analyzing the secondary-structure features of TSEs in protein folding [77], structure modeling [78,79], and other molecular dynamics simulations [80]. Here, in order to simplify the analysis, we modify Equation (4a), as follows,

$$a_{ij}=\{\begin{array}{cc} 1 & \mathrm{if}\;\mathrm{residue}\;i,j\;\mathrm{form}\;a\;\mathrm{native}\;\mathrm{contact}\\ 0 & \mathrm{else} \end{array},$$

and define contact-map of TSEs through ensemble average,

(4b) $$c_{ij}=\langle a_{ij}\rangle {}^{\mathrm{TSE}}$$

Contact maps measure the probability that two residues tend to stay together as in their native states, which can be interpreted as a two-dimensional (2D) projection of the tertiary structures.

## 4. Conclusions

It had been recognized that non-native contact interactions could play an important role in shaping protein folding transition states, thus understanding the energetic-frustration effects caused by these interactions might be an important theoretical task. Here, we examined energetic frustrations in all-*β* structures by comparing the folding mechanisms of 9 homologous Ig-like *β*-sandwich domains. The studied domains share the same complex Greek-key topology and have similar tertiary structures, thus topological frustrations in the folding dynamics are minimized. Two sets of transition state ensembles were obtained through variable temperature simulations using the minimal and the frustrated Gō-like models, respectively. Energetic frustrations were modeled with non-native hydrophobic interactions using the standard Lennard-Jones potential function. The selected domains share the same sequence, except for a dominant single hydrophilic-hydrophobic mutation among them, thus a sequence-alignment arrangement might locate energetic frustrations in the 3D structures. Our calculations suggest that energetic frustrations cause highly heterogeneous effects on the folding of *β*-sandwich structures, as illustrated by a diverse change distribution in the folding of secondary structure elements and in correlations between folding-patches in neighboring patch-pairs. Our results also show that *β*-sandwich structure exhibits a high resistance to a variety of energetic frustrations in keeping its hydrophobic core in the nucleation-condensation folding.

Unlike the cases in folding of all-*α* proteins [51], beta-structure energetic frustrations tend to stabilize the transition states; this might be ascribed to the competition between the native hydrophobic contacts and nonnative hydrophobic contacts in energetic frustrations. In this sense, energetic frustrations are also closely related with the native-contact networks of *β*-sandwich structures in reshaping the protein-folding dynamics. When considering that both *β*;-sandwich domains and all-*α* domains are all small and tightly-compact single-domain structures, their consistency in the interplay between energetic frustrations and native contact networks seems not difficult to appreciate [36]. However, topological differences between *β*- and *α*-structures do have influence in formation of native-contact networks: most edges in alpha-helical structures are formed by a sequentially neighboring residue-pair, in which one N–H group orients toward another C=O group in a direction that is largely parallel to the helical axis, while those in beta-structures mostly link sequentially distant residues and the hydrogen bonds is usually perpendicular to the beta strands themselves. Such differences in secondary-structure arrangement makes energetic frustrations in *β*-sandwich proteins more heterogeneous when compared with those in helical structures, thus the formation of *β*-sheet in the *β*-sandwich might feel more frustrated than does the *α*-helix in all-*α* structures. Particularly, nonnative energetic frustrations that are caused by single site mutation in linkers L3 and L2 introduce large folding fluctuations in secondary structures, and alter the folding correlations between different folding patches. These results suggest that the folding process of *β*-sandwich structures might be tuned by carefully manipulating nonnative energetic frustrations at the residue level.

## Figures and Tables

**Table 1:** Backbone/*C*_α_ root mean square distance (in Å) between selected Ig-like *β*-sandwich domains.

Protein	HC19	R23A	N36I	A80G	Y94D
HC19	0				
R23A	0.62/0.51	0			
N36I	0.53/0.47	0.58/0.48	0		
A80G	0.57/0.52	0.61/0.54	0.53/0.49	0	
Y94D	0.76/0.79	0.77/0.73	0.65/0.62	0.69/0.64	0

## Supplementary Materials

Supplementary materials can be found at http://www.mdpi.com/1422-0067/19/5/1515/s1.
