# Supplementary material for: Nonnative Energetic Frustrations in Protein Folding at Residual Level: A Simulation Study of Homologous Immunoglobulin-like β-Sandwich Proteins
Source: Int J Mol Sci. 2018 May 18;19(5):1515. doi: 10.3390/ijms19051515 (PMC5983731; doi:10.3390/ijms19051515)

supplementary materials

Nonnative energetic frustrations in protein folding at residual level: a simulation study of homologous immunoglobulin-like β-sandwich proteins

Running title: Energetic frustrations in β-sandwich proteins

Key words: nonnative energetic frustration; non-native contact; frustrated Gō-like model; β-sandwich protein; hydrophilic-hydrophobic mutation

Authors: Yunxiang Sun[a,b] Feng Ding[b] and Dengming Ming[a*]

[a] College of Biotechnology and Pharmaceutical Engineering, Nanjing Tech University, 30 Puzhu South Road, Nanjing, Jiangsu 211816, PR China, [b] Department of Physics and Astronomy, Clemson University, Clemson, South Carolina 29634, USA

[*]Contact information:
Dengming Ming
Biotech Building Room B1-404
College of Biotechnology and Pharmaceutical Engineering,
Nanjing Tech University,
30 South Puzhu Road
Jiangsu 211816, PR China
Tel: 8625-58139942
Email: dming@njtech.edu.cn

Figure S1. Compare the conventional constant temperature folding (CTF) simulation with that of Variable Temperature Folding (VTF) simulation. A) CTF simulation of all-β sandwich domains HC19, B) VTF simulation of HC19, C) the free energy distribution of the two simulations as log inverse of distribution probability. Compared with CTF, VTF experiences double transition between the two states within the same number of simulation steps, and the resulted free energy landscape has a wider sampling distribution.

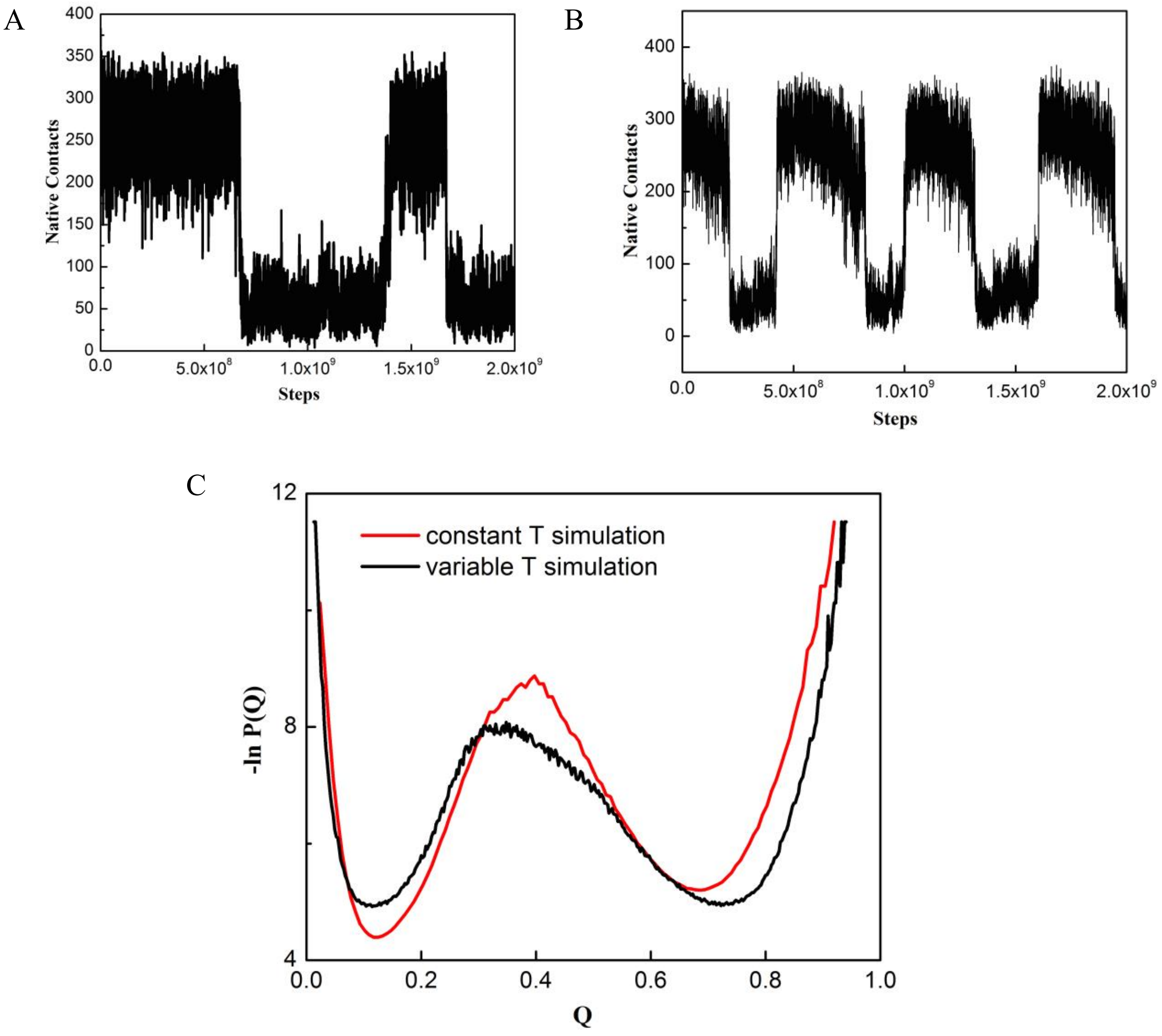

Figure S2. Comparison of residual ϕ-values (black) calculated using the conventional Gō-like minimal model and those (red) using the frustrated Gō-like model for the 9 β-sandwich domains that have the same sequence except a single mutation; the difference in residual ϕ-value changes reveal, to some extent, how the different local environment of mutation-site affects the folding dynamics of the β-sandwich structure.

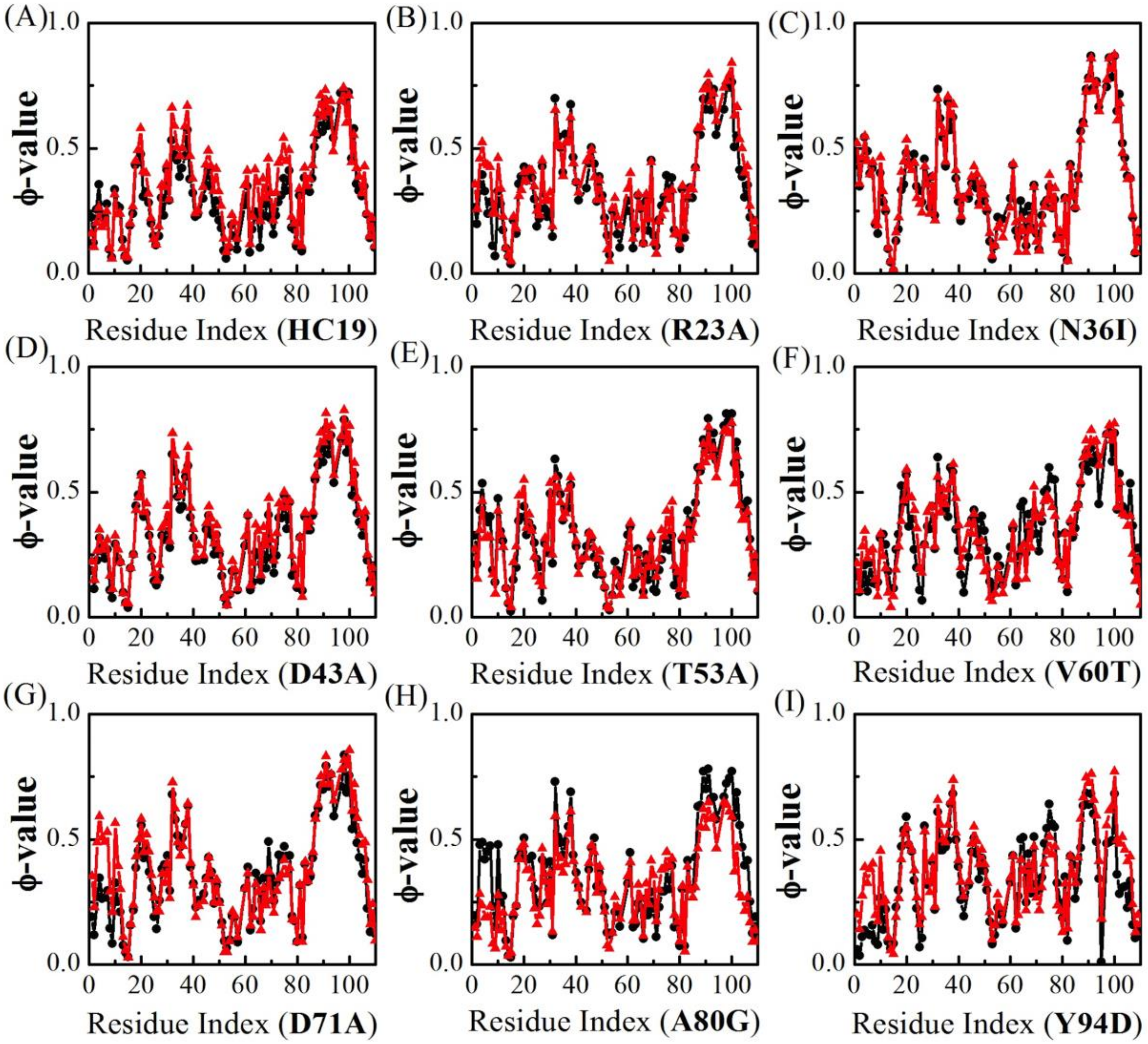

Figure S3. Comparison experimental residual ϕ-values with simulated ϕ-values for HC19 domain. The experimental residue index is derived through sequence-alignment.

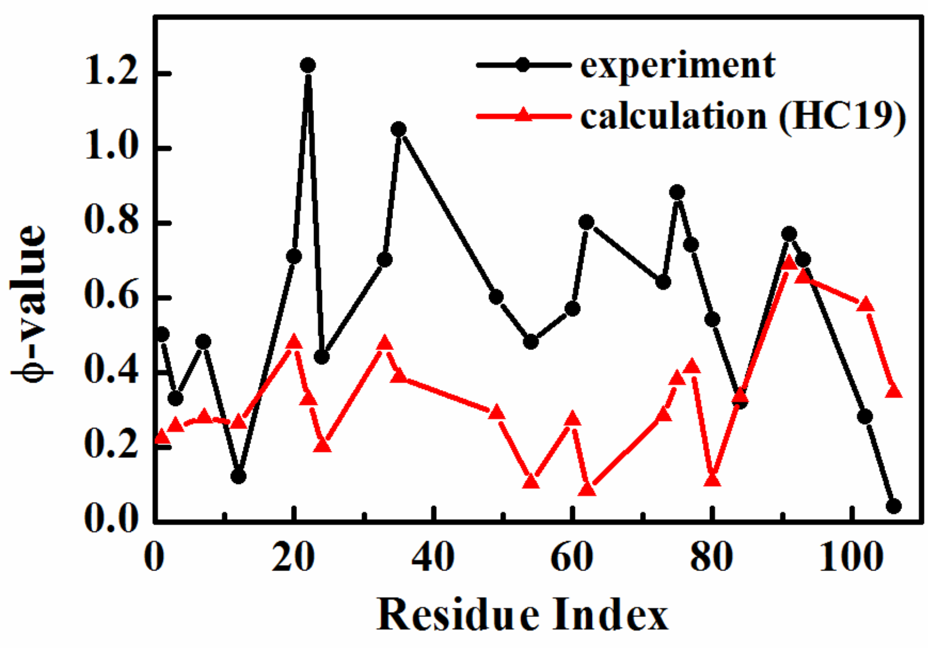

Figure S4. The contact maps of transition states for the 9 β-sandwich domains. In each map, the upper left triangle is derived from the frustrated Gō-like model which is compared with the lower right triangle derived from the conventional Gō-like minimal model.

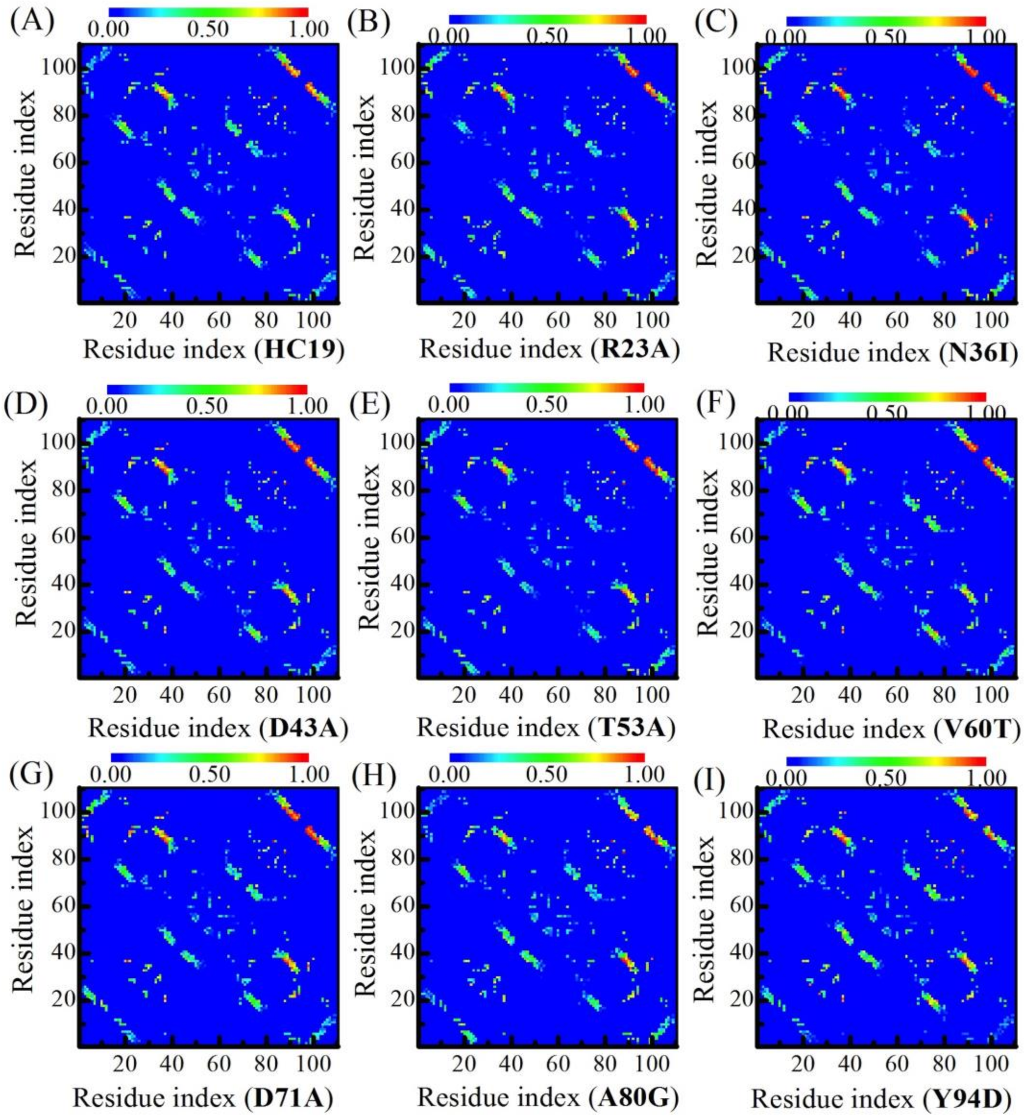

Supplement: Supplementary file 1 [file ijms-19-01515-s001.pdf]
